# Analysis of risk factors for duodenal leak after repair of a duodenal perforation

**DOI:** 10.1186/s12893-023-02005-7

**Published:** 2023-05-10

**Authors:** Jianping Liu, Song Zhou, Shaoyi Wang, Xiaojun Xue

**Affiliations:** Department of General Surgery, Dongnan Hospital of Xiamen University, School of Medicine, Xiamen University, No.269, Zhanghua Road, Xiangcheng District, Fujian Prov Zhangzhou, China

**Keywords:** Repair of duodenal perforation, Duodenal leak, Risk factors, Logistic regression, ROC curve

## Abstract

**Background:**

Repairing of a duodenal perforation is a well accepted procedure, but clinically, approximately 4% of patients develop duodenal leaks after perforation repair, increasing the risk of death. We retrospectively analyzed clinical data from 168 patients at our hospital to explore risk factors for duodenal leak after perforation repair and developed a nomogram for predicting postoperative duodenal leak.

**Methods:**

This retrospective case–control study totalled 168 patients undergoing repair of a duodenal perforation with omentopexy at the General Surgery Department, Dongnan Hospital of Xiamen University, from January 2012 to January 2022. The patients were divided into the non-leak group and the leak group. Risk factors were evaluated by analyzing the patient’s sex, shock, diameter and anatomic position of the ulcer, use of NSAIDS and Glucocorticoid, history of drinking, diabetes, chronic diseases, age, time of onset of symptoms and lab tests.

**Result:**

One hundred fifty-six patients (92.9%) who did not develop leaks after repair of a duodenal perforation were included in the non-leak group, and 12 (7.1%) developed leaks were included in the leak group. In univariate analysis, there were significant differences between the two groups referring to age, shock, NSAIDs, albumin, and perforation size (*P* < 0.05). The area under the ROC curve for perforation diameter was 0.737, the p-value was 0.006, the optimal cutoff point was 11.5, sensitivity was 58.3%, and specificity was 93.6%, the positive predictive value is 41.1%, and the negative predictive value is 98.0%. In the internal validation of the performance of the nomogram, the C-index and AUC of the model were 0.896(95%CI 0.81–0.98), demonstrating that the nomogram model was well calibrated.

**Conclusion:**

The study discussed the risk factors for postoperative duodenal leak in patients undergoing repair of a duodenal perforation, and a nomogram was constructed to predict the leak. Future prospective studies with large sample sizes and multiple centres are needed to further elucidate the risk of duodenal leak after repair of a duodenal perforation.

## Background

Peptic ulcer disease is common, with a lifetime prevalence in the general population of about 5–10%, with an annual incidence of 0.1–0.3% [1]. The main clinical manifestations are abdominal pain, hematemesis, and black stools. Perforation, as a complication of peptic ulcer disease, is a well-known complication of surgical acute abdomen. The incidence rate in females is higher, and the perforation of peptic ulcers is most common in the duodenal bulb [2]. Peptic ulcer perforation(PUP) is the second most common ulcer complication after bleeding [3]. At the end of the last century and the beginning of this century, with the discovery of HP infection and the application of H2 receptor antagonists and proton pump inhibitors, it is no longer challenging to cure ulcers. Generally, an excellent therapeutic effect can be achieved after surgical intervention. Now, the first choice for PUP is simple perforation repair. Although the surgery is effective, this operation cannot cure ulcers. If no regular treatment is received after surgery, complications such as perforation and bleeding will still occur [4]. Clinically, placement of a drain near the duodenal repair or placement of a jejunal feeding tube may help to decrease the severity or development of a postoperative duodenal leak [5, 6].

Nevertheless, 4% of patients still suffer from the duodenal leak after perforation repair [7]. The primary manifestation is abdominal diffuse peritonitis which increases the risk of death. In this study, we analyzed the associated risk factors for postoperative duodenal leak and constructed a nomogram for predicting the leak.

## Methods

### Study design

This study is a retrospective analysis, and data were permitted by the Institutional Review Board of Dongnan Hospital of Xiamen University. The study complied with guidelines outlined under the Consolidated Standards of Reporting Trials (CONSORT) checklist.

### Patients

Data acquisition was performed using a retrospective case–control study approach. We retrospectively collected the clinical data from 168 patients who underwent repair of a duodenal perforation with omentopexy at the General Surgery Department, Dongnan Hospital of Xiamen University, from January 2012 to January 2022. The cases were screened according to the below inclusion and exclusion criteria. Ultimately, 168 patients were included in the study, 138 males and 30 females, with a median age of 61 years (11–88).

Currently, there are no guidelines or consensus literature on the diagnosis of duodenal leak. However, many scholars believe that the duodenal leak is suspected in the following circumstances—①Repeated chills and fever. ②Para-duodenal abscess collection. ③Persistent discharge of gastric or intestinal fluid into the drainage tube. ④Abnormal extravasation or duodenal leak observed during gastroenterography, gastroscope, or surgery [8–11]. According to the criteria, we divided the 168 patients into a non-leak group and a leak group, with the non-leak group of 156 patients and the leak group of 12 patients. We compared the two groups' data (sex, shock, diameter and anatomic position of the ulcer, use of NSAIDS and Glucocorticoid, history of drinking, diabetes, chronic diseases, age, time of onset of symptoms and lab tests) to analyse the associated risk factors and develop a nomogram predict model. We defined *shock* as blood pressure of less than 90/60 mmHg on admission or a drop of 30 mmHg from the baseline.

### Inclusion and exclusion criteria

Inclusion criteria (1) History of gastroduodenal ulcer. (2) Abdominal X-ray and CT show abdominal free gas and other signs of ulcer perforation [12]. (3) Patients who have undergone suture repair of the duodenal perforation with omentopexy. (4) All patients with duodenal leak developed in the postoperative during hospitalization. (5) Complete case data. Exclusion criteria (1) Incomplete case data. (2) Patients with a history of malignancy. (3) Patients with other digestive tract diseases.

### Statistical analysis

Statistical analysis was performed using SPSS version 24.0 and R version 3.5.3. The χ2 test or Fisher's exact test was employed for count data and the t-test for measurement data. A univariate analysis was conducted to identify factors associated with postoperative duodenal leak. Based on the results, a nomogram was developed to predict the likelihood of duodenal leak. The model can be visualized using a nomogram. Additionally, its accuracy was assessed through internal validation. (*C*-index, ROC,bootstrap method). *P* < 0.05 indicates a statistically significant difference.

## Results

One hundred sixty-eight cases were included into this study. 156 (92.9%) who did not develop leaks after repair of a duodenal perforation were included in the non-leak group and 12 (7.1%) who developed leaks were included in the leak group. In the leak group, the mean age was 69 ± 16 years, mean onset time of symptoms was 25 ± 24 h, of which 8 (66.7%) were male, and 4 (33.3%) were female. Six patients (50%) had shock before surgery, 5 (42%) used NSAIDs, none used glucocorticoids, 2 (16.7%) had a history of alcohol consumption, none had a history of diabetes, and 5 (41.7%) had other chronic diseases (hypertension, coronary heart disease, hepatic cirrhosis, chronic renal Insufficiencies). The preoperative laboratory tests of the 12 patients with duodenal leak were HGB 127 ± 25 g/L, ALB 29 ± 7 g/L, WBC 11 ± 7 × 10^9^/L, PLT 283 ± 101 × 10^9^/L, and all 12 patients had perforations in the anterior wall of the duodenal bulb. The mean perforation diameter was 10 ± 4 mm. Univariate analysis of the patients' data showed significant differences between the two groups in age, shock, NSAIDs, albumin, and perforation diameter (all *P* < 0.05) (Table 1).

**Table 1:** Univariate analysis of duodenal leak after repair of a duodenal perforation [*x* ± *s,n* (%)]

Patient characteristics	Non-leak (156)	Leak (12)	Inspection-value	*P*-value
Sex				
M	130	8	1.127	0.288
F	26	4		
Shock				
Y	12	6	16.661	0.000
N	144	6		
Anatomic position of the ulcer				
Anterior wall of duodenal bulb	146	12	0.818	0.664
Posterior wall of duodenal bulb	5	0		
Descending duodenum	5	0		
NSAIDs				
Y	16	5	7.385	0.007
N	140	7		
Glucocorticoid				
Y	10	0	0.074	0.786
N	146	12		
History of drinking				
Y	17	2	0.018	0.893
N	139	10		
Diabetes				
Y	19	0	0.657	0.418
N	137	12		
Other chronic diseases				
Y	55	5	0.018	0.893
N	101	7		
Age,mean (*SD*),years	59 ± 16	69 ± 16	-2.175	0.031
HGB (g/L)	129 ± 25	127 ± 25	0.334	0.739
ALB (g/L)	39 ± 7	29 ± 7	5.166	0.000
Perforation diameter, mean (*SD*), mm	6 ± 4	10 ± 4	-3.692	0.000
Mean onset time of symptoms, (*SD*), min	18 ± 24	25 ± 24	-0.862	0.387
WBC (10^9^/L)	14 ± 7	11 ± 7	1.716	0.088
PLT (g/L)	253 ± 100	283 ± 101	-0.817	0.316

**Fig. 1:**
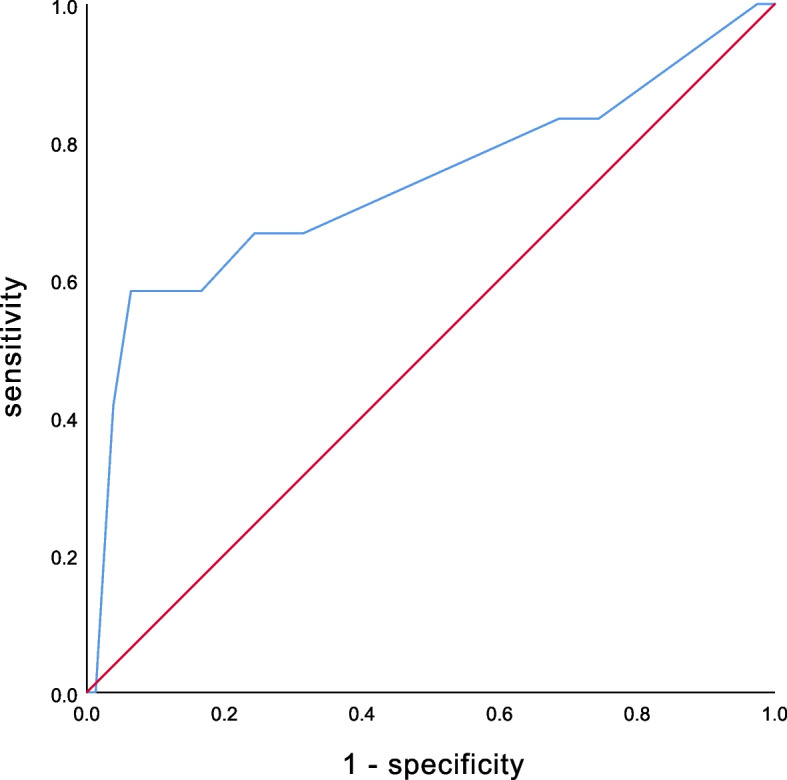
ROC curve for perforation diameter

ROC Curve Analysis: Perforation diameter was analyzed as an important risk factor in univariate analysis. The area under the ROC curve for perforation diameter was 0.737, *p*-value 0.006, 95% confidence interval (0.554, 0.921). When the cutoff value for perforation diameter was set at 11.5, the sensitivity and specificity were 58.3% and 93.6%, the positive predictive value is 41.1%, and the negative predictive value is 98.0%. (Table 2) (Fig. 1).

**Table 2:** ROC analysis of perforation diameter in the diagnosis of duodenal leak

Related factors	AUC	*P*-value	Cut-off value	Sensitivity	Specificity	Positive predictive value	Negative predictive value
Perforation diameter	0.737 (0.554–0.921)	0.006	11.5	0.583	0.936	0.411	0.980

**Fig. 2:**
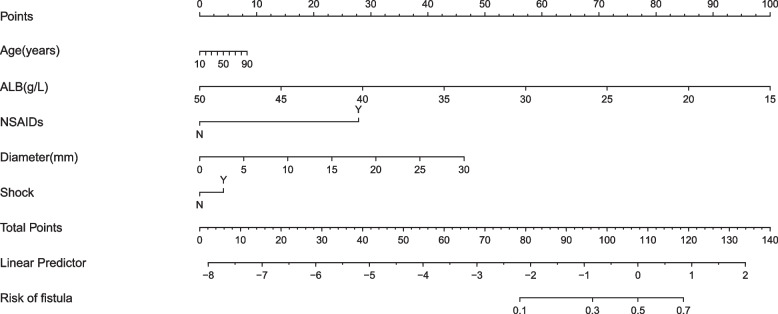
Nomogram to predict duodenal leak after repair of a duodenal perforation

**Fig. 3:**
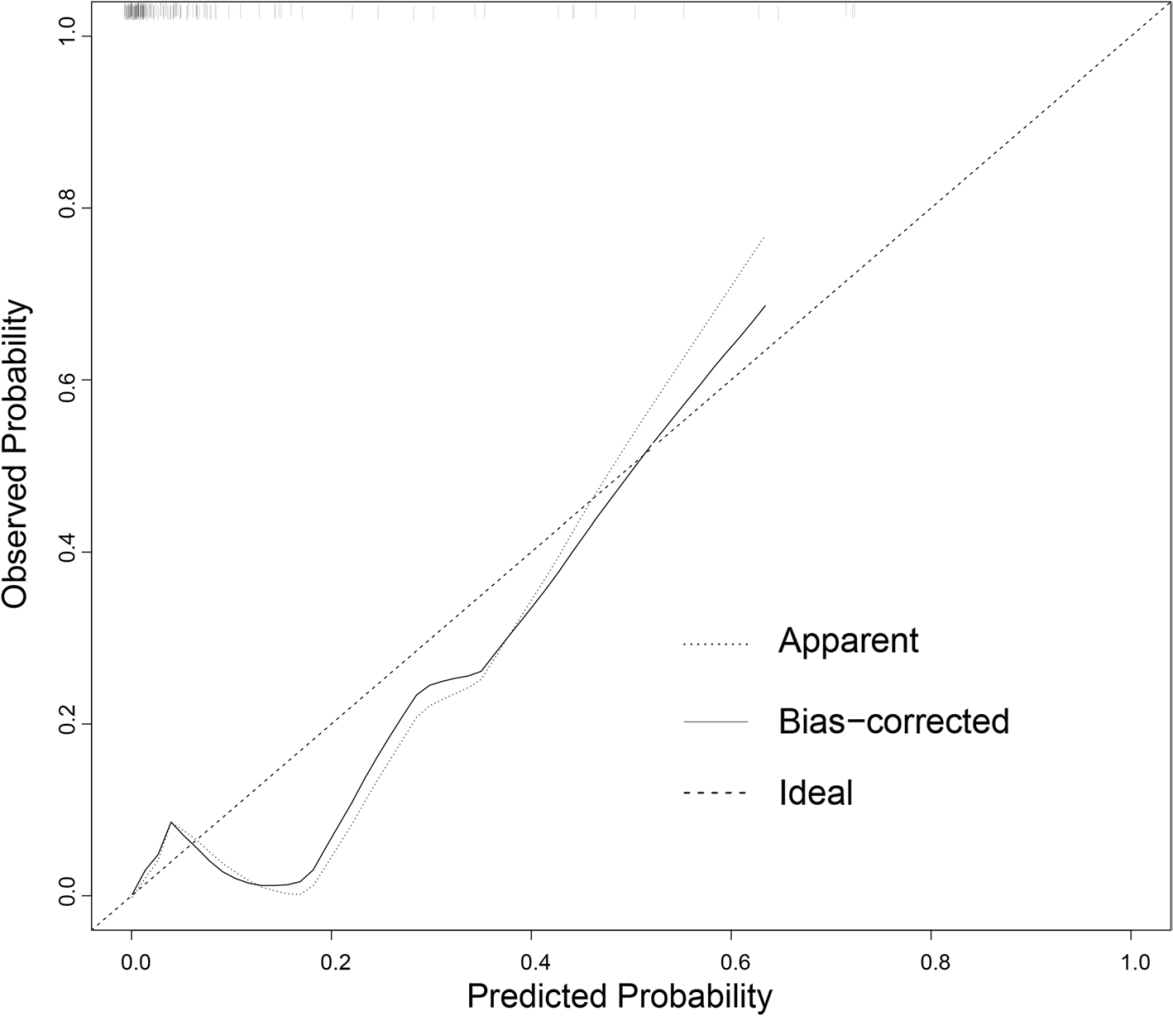
Calibration plot of the nomogram for the probability of duodenal leak after repair of a duodenal perforation

Nomogram establishment: The nomogram prediction model was developed based on the factors that showed statistically significant differences in the univariate analysis. The value of each factor in Fig. 2 corresponds to the "score" of the first row of the nomogram. Each factor's individual score is added to obtain the total score. The total score of the nomogram is used to evaluate the probability of the duodenal leak, and the higher the score, the greater the chance of the duodenal leak. In the internal validation of the performance of the nomogram, the *C*-index and AUC of the model were 0.896(95%*CI* 0.81–0.98), which shows that the nomogram model was well calibrated (Figs. 2 and 3).

## Discussion

Gastroduodenal ulcers are a common duodenal disorder. Its most serious complication is ulcer perforation. With the advent of proton pump inhibitors(PPIs) and drug combination therapy against H. pylori, the effectiveness of medical treatment of gastroduodenal ulcers has greatly improved [13, 14]. As a result, surgical treatment for perforation has evolved from gastric subtotal gastrectomy for the ulcer itself to simple perforation repair [15]. Although the majority of patients who undergo repair of a duodenal perforation recovered well, 4% of patients still develop a postoperative duodenal leak [3]. After performing univariate analysis of clinical data from 168 patients this study revealed that the preoperative values of age, shock, NSAIDs, albumin, and intraoperative perforation diameter were risk factors for duodenal leak after repair of a duodenal perforation. A preoperative predictive model also including the intraoperative diameter of the perforation was developed by combining the five clinical risk factors mentioned above to estimate the risk of postoperative duodenal leak in patients who undergo repair of duodenal perforation. In the patients in this study, the mean age of the leak group was higher than that of the non-leak group. The reason for poor postoperative recovery may be the low self-resistance and immunity of the elderly [16]. The study by Lunevicius et al. showed that shock is an independent risk factor affecting the prognosis of repair of a duodenal perforation, while the analysis by Irwin considered shock to be an independent risk factor only in patients older than 70 years [17, 18]. In this study, univariate analysis showed a correlation between shock and postoperative duodenal leak, suggesting the need to improve the patient's shock status as much as possible before surgery. The primary mechanism of NSAIDs is to stimulate gastric acid secretion and decrease the blood supply of gastric mucosa by inhibiting prostaglandins. Therefore, excessive use of NSAIDs can damage the gastroduodenal mucosa, resulting in duodenal mucosa erosion, ulceration and even perforation of the wall [19, 20]. Our study revealed a very important finding that patients taking NSAIDs, especially in the elderly are more likely to develop leaks after perforation repair because of the erosions and ulcers on the duodenal wall at the time of mucosal injury due to the drug effects described above.

The study noted that two important risk factors, albumin and perforation diameter, have received little attention in previous studies on duodenal leaks after perforation repair. Ishida et al. considered albumin related to a nonbacterial inflammatory response and found that albumin is highly and negatively correlated with inflammation [21]. Dubniks et al. found that albumin was associated withmaintenance of the inner wall of the vessel and the integrity of the vascular endothelial surface, and they believe that the vascular endothelial surface is an essential structural material that prevents fluid extravasation [22]. Combined with the results of this study, low albumin levels are associated with aseptic inflammatory reactions and tissue oedema at the suture site after perforation repair. When serum albumin levels decrease, the osmotic pressure can be abnormal, resulting in a large amount of fluid extravasation, causing edema. It will weaken the healing ability of body tissues which increases the risk of postoperative duodenal leak [23]. Therefore, providing perioperative nutritional support to patients with hypoalbuminemia may have a certain significance in reducing the occurrence of postoperative leaks [24].

Perforation diameter as an risk factor for postoperative leak. The larger the perforation diameter, the more severe the damage to the intestinal mucosa and the higher the risk of the leak. Secondly, if the tissue around the perforation is fragile and stiff, the tension at the suture line is also an important reason for perforation. In the ROC analysis, the AUC was 0.737, and the sensitivity and specificity were 0.583 and 0.936, respectively, when the cutoff value was 11.5 mm. This indicates that leak is more likely to occur when the perforation diameter is 11.5 mm or greater. For patients with a perforation diameter of 11.5 mm or greater, we can consider other surgical techniques. Omental plugging technique is a suitable alternative with less incidence of leakage, shorter procedural time, and easy to perform. Duodenal (pyloric exclusion) with primary repair is also an effective and feasible procedure [25]. Also others have described using a loop of jejunum as an aerosol patch or even bringing up a Roux-en-Y limb and performing a diode o-jejunal anastomosis for giant duodenal perforations of > 2 cm. In addition, attention should be paid to the development of postoperative leaks, such as placement of a drain near the duodenal repair or placement of a jejunal feeding tube may help to decrease the severity or development of a postoperative duodenal fistula [5, 6].

### Limitation

However, the study still has several limitations. Our results were established as a retrospective study, thus there will be unavoidable selection bias including a small sample size and being a single-site study. In addition, the mechanism responsible for the risk factors of the fistual requires further exploration and clarifcation.

## Conclusion

In conclusion, we discussed the risk factors for postoperative duodenal leak in patients undergoing repair of a duodenal perforation. A nomogram was constructed that incorporated the five significant factors, including age, shock, NSAIDs, albumin, and perforation diameter. This predictive model estimates the probability of patients developing a duodenal leak after undergoing duodenal perforation repair. If the predicted probability of a leak is high, corresponding intervention measures such as intravenous supplementation of albumin, changing the surgical approach, placing a drain tube, and placing an enteral nutrition tube for early enteral nutrition may be necessary. These interventions can to some extent prevent the occurrence of postoperative leaks and improve the patient's postoperative recovery.

## Data Availability

The data sets generated and analyzed in this study are not publicly available due to potential invasion of personal privacy but are available from the corresponding author upon reasonable request.

## Abbreviations

NSAIDs: Nonsteroidal anti-inflammatory drugs
HP: Helicobacter pylori
PUP: Peptic ulcer perforation
CT: Computed tomography
HGB: Hemoglobin
ALB: Albumin
WBC: White blood cell count
PLT: Platelet
PPIs: Proton pump inhibitors
SD: Standard deviation
ROC: Receiver operating characteristic curve
AUC: Area under the curve
